# Carbon Dots in Treatment of Pediatric Brain Tumors: Past, Present, and Future Directions

**DOI:** 10.3390/ijms24119562

**Published:** 2023-05-31

**Authors:** Frederic A. Vallejo, Ganesh Sigdel, Eduardo A. Veliz, Roger M. Leblanc, Steven Vanni, Regina M. Graham

**Affiliations:** 1 Department of Neurosurgery, Miller School of Medicine, University of Miami, 1095 NW 14th Terrace, Miami, FL 33136, USA; 2 Department of Chemistry, University of Miami, 1301 Memorial Drive, Coral Gables, FL 33146, USA; 3 HCA Florida University Hospital, 3476 S University Dr., Davie, FL 33328, USA; 4 Department of Medicine, Dr. Kiran C. Patel College of Allopathic Medicine, Davie, FL 33328, USA; 5 Sylvester Comprehensive Cancer Center, University of Miami Health System, Miami, FL 33136, USA

**Keywords:** pediatric brain tumor, precision medicine, nanoparticle, nanomedicine, cancer, carbon dot

## Abstract

Pediatric brain tumors remain a significant source of morbidity and mortality. Though developments have been made in treating these malignancies, the blood–brain barrier, intra- and inter-tumoral heterogeneity, and therapeutic toxicity pose challenges to improving outcomes. Varying types of nanoparticles, including metallic, organic, and micellar molecules of varying structures and compositions, have been investigated as a potential therapy to circumvent some of these inherent challenges. Carbon dots (CDs) have recently gained popularity as a novel nanoparticle with theranostic properties. This carbon-based modality is highly modifiable, allowing for conjugation to drugs, as well as tumor-specific ligands in an effort to more effectively target cancerous cells and reduce peripheral toxicity. CDs are being studied pre-clinically. The ClinicalTrials.gov site was queried using the search terms: brain tumor and nanoparticle, liposome, micelle, dendrimer, quantum dot, or carbon dot. At the time of this review, 36 studies were found, 6 of which included pediatric patients. Two of the six studies investigated nanoparticle drug formulations, whereas the other four studies were on varying liposomal nanoparticle formulations for the treatment of pediatric brain tumors. Here, we reviewed the context of CDs within the broader realm of nanoparticles, their development, promising pre-clinical potential, and proposed future translational utility.

## 1. Introduction

Brain tumors are a significant source of morbidity and mortality within the pediatric population [1]. The Central Brain tumor Registry of the United States estimates that 5260 new cases of both malignant and non-malignant tumors of the central nervous system (CNS) will be diagnosed in children ages 0–19 during 2023 alone. Between 2014 and 2018, there were an average of 539 deaths annually in children and adolescents aged 0–19 due to tumors of the brain and CNS. Gliomas represented the majority of all CNS tumors at just above forty percent, followed by tumors of the pituitary comprising more than fourteen percent, and finally, embryonal tumors representing just under ten percent [2]. The other approximately thirty-five percent of tumors were comprised of many groups of less-common tumors. Roughly forty-thousand children were noted to be living with a diagnosed CNS tumor during 2022. Though the prevalence of pediatric leukemia was also noted to be roughly forty-thousand, the recent advancements in the treatment of hematologic malignancies have not been paralleled in the treatment of pediatric brain and CNS tumors [3]. These pediatric patients often face extremely difficult treatment regimens and often succumb to these devastating pathologies. Additionally, pediatric drug development garners much less attention than adult drug development, resulting in fewer appropriately labeled pediatric drugs [4]. Therefore, expeditious development, refinement, and assessment of novel treatment modalities are necessary to effectively combat these malignancies in the future.

Many efforts have been undertaken to investigate novel therapies to circumvent many of the challenges confronted in the treatment of pediatric brain tumors including, but not limited to the blood–brain barrier, inter- and intra-tumoral heterogeneity, the development of drug resistance, and tumor invasion into surrounding neurocritical structures [5]. The blood–brain barrier (BBB) has been extensively characterized and studied [6,7,8]. This network of pericytes and podocytes acts to selectively inhibit certain compounds and molecules from passing into the brain itself, thereby limiting the ability for certain drugs to reach their target. In addition to the selective permeability of the blood–brain barrier, aggressive tumors have been shown to modify their expression profiles in response to certain drugs, upregulating membrane pumps to remove cytotoxic therapies and silencing pro-apoptotic cascades that would otherwise exist in normal tissue. Additionally, tumors can evolve over time as susceptible cell populations are eradicated, and those that have favorably mutated to resist the intervention survive and proliferate.

Several of the most-common pediatric brain tumors including pilocytic astrocytomas, medulloblastomas, ependymomas, and high-grade gliomas (HGGs) including diffuse intrinsic pontine gliomas (DIPGs), or diffuse midline glioma, and glioblastoma (GBM) are commonly found in extremely delicate areas of the brain. Both pilocytic astrocytomas and medulloblastomas commonly arise in the posterior fossa, where growth may result in severe cerebellar dysfunction, aqueduct obstruction and ensuing non-communicating hydrocephalus, herniation, and drop-metastases in the setting of advanced disease. Ependymomas arise along the lining of the ventricular system, commonly arising in the fourth ventricle and central canal, where expansion may result in increased intracranial pressure and compression of surrounding structures. DIPGs, as their name suggests, are found within the brainstem at the pons. These tumors are extremely difficult to treat, and their growth commonly results in marked cranial nerve deficits, long tract signs, and ensuing critical brainstem function failure. GBMs grow extremely quickly with cells invading sites distant from the tumor’s site of origin, underscoring the difficulty in achieving complete resections in these patients. Surgical resection remains a primary aim for many of these tumors with subsequent radiotherapy and or chemotherapy in certain cases depending on the pathology. Other tumors, such as DIPGs, are rarely considered candidates for surgical resection, and contemporary treatment paradigms are insufficient to improve pediatric patient prognoses.

For over a decade, effort has been aimed to minimize therapeutic toxicity in favorable-risk tumor cohorts while attempting to improve outcomes and discover novel modalities in poor-risk tumor groups [9]. Improvements in genomic and proteomic analysis have rendered thorough descriptions of the expression landscape across several pediatric brain tumor types, leading to more thorough classifications of tumor sub-types [10,11]. By utilizing these data, researchers can investigate therapies targeted to the precise molecular and genomic susceptibility of unique tumor groups.

Nanoparticles have been investigated as novel drug delivery systems by which conventional therapy may be improved or modified. The term “nanoparticle” is an umbrella term that encompasses metallic, organic, and micellar molecules of varying structures and compositions. Broadly, nanoparticles refer to particles ranging from 1 to 100 nanometers in size with varying biological, chemical, and physical properties. The more commonly studied groups of nanoparticles, which have been investigated in preclinical studies, include liposomes, dendrimers, metallic nanoparticles, quantum dots, and carbon dots (CDs). These categories of nanoparticles differ in their composition, preparation method, ability to enter cells, toxicity profile, and proposed translational utility. Herein, we posit that CDs are a novel and promising theranostic modality, which can be extensively modified for targeted drug delivery systems, as well as employed for tumor imaging. The aim of this review was to provide an introduction to pediatric brain tumors and CDs, discuss the relevant published literature, and propose a path forward for the use of CDs as a novel drug delivery platform in the treatment of pediatric brain tumors.

## 2. Types of Nanoparticles

Liposomes are spherical lipid bilayers with inner aqueous compartments, whereas micelles consist of a singular layer, which can encapsulate hydrophobic therapies. Both have been employed as drug delivery platforms, encapsulating drugs that otherwise may not readily enter hydrophobic environments. Dendrimers are three-dimensional polymers with tightly controlled nanoarchitectonic properties, enabling them to have specific functional groups exposed at the surface for gene therapy and drug delivery. Quantum dots are nanoparticles with strong fluorescent properties, which have been under investigation as a diagnostic imaging modality. Metallic nanoparticles most commonly refer to those comprised of gold, silver, copper, iron, as well as zinc oxide, which may be used to deliver drugs, as well as for their intrinsic tumoricidal and magnetic capabilities.

In a recent comprehensive review of nanoparticles for use in pediatric brain tumors, each of the aforementioned categories was summarized with several strengths and weaknesses demonstrated across several preclinical studies [12]. For instance, metallic nanoparticles offer the advantage of harboring anti-cancerous properties, potential sensitization to radiotherapy, and the ability to induce magnetic hyperthermia, but have the disadvantage of necessitating costly precursors, as well as peripheral accumulation and cytotoxicity. Lipid nanomaterials are easily assembled and biocompatible, but have a high uptake in the liver and spleen. The surface moieties on dendrimer nanoparticles can be tightly controlled to load certain compounds, but may result in potential neurotoxicity. Finally, quantum dots are highly florescent and can, therefore, be used for photodynamic therapy, but employ heavy metals such as cadmium with complex formulations.

## 3. Pediatric Nanoparticle Clinical Trials

A query of ClinicalTrials.gov with the search terms brain tumor and nanoparticle or liposome, micelle, dendrimer, quantum dot, or carbon dot revealed 36 studies in total. Of those 36 studies, only six included pediatric patients. Two of the six studies were on convection enhanced delivery of MTX110, a Panobinostat nanoparticle formulation, in the treatment of midline gliomas or DIPGs. The other four studies were on varying liposomal nanoparticle formulations including liposomal doxorubicin, Marquibo, which is Vincristine Sulfate in a liposomal package, liposomal cytarabine, and liposomal irinotecan for the treatment of pediatric brain tumors. At the time of this study, results had only been posted for one of the six studies in which seven patients had been enrolled (NCT03566199). This phase I study demonstrated that the Convection Enhanced Delivery (CED) of Panobinostat produced a serious adverse effect of R-sided musculoskeletal weakness and vagus nerve disorder in 1/7 patients. Additionally, 85.7% of the participants were alive at 12 months after diagnosis (95% CI 63.3 to 100). Notably, all studies were phase I-phase I/II clinical trials. At the time of this review, no studies were available on CDs in pediatric brain tumors.

## 4. Carbon Dots

**Figure 1:**
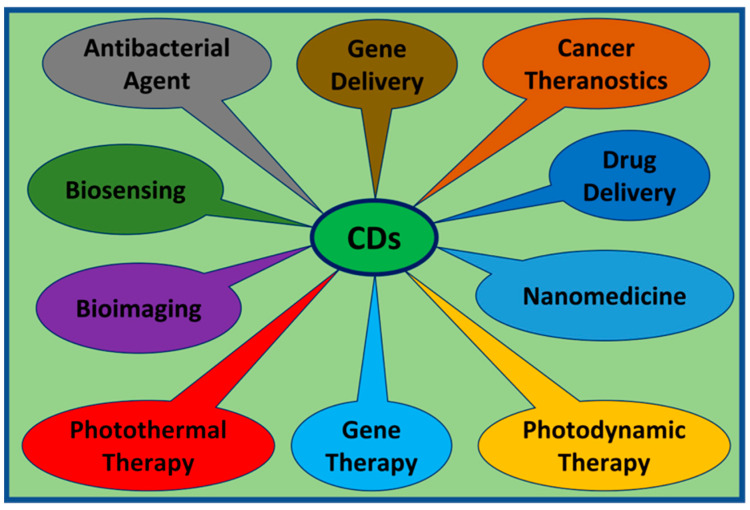
CDs are currently being investigated on multiple fronts including: gene delivery, theranostics, drug delivery, nanotherapeutics, photodynamics, photothermal therapy, bioimaging, biosensing, and as antibacterial agents.

CDs are a new class of fluorescent and zero-dimensional (0D) carbon nanomaterials typically <10 nm in size. They have been studied extensively since their initial description in 2004 [13]. The identification of CDs was serendipitous when “fluorescent impurities” were observed during the synthesis of carbon nanotubes. Subsequently, an explosion of research pertaining to their synthesis, properties, and applications has occurred. CDs are analogous to semiconductor quantum dots in regard to size and photoluminescent properties, but unlike quantum dots, which contain heavy metals, CDs are largely non-toxic [14]. CDs demonstrate good water dispersibility and high bio-compatibility [15]. In addition, CDs’ tunable fluorescent properties and abundant surface functional groups allow for easy modifications [16]. Combined with the low cost of synthesis, eco friendliness, and good stability, CDs have attracted research focused on optical, energy, and bio-medical applications [14]. The biomedical uses of CDs have been studied extensively in recent years. CDs can be used in various biomedical applications such as bioimaging [17,18], nanomedicine, drug delivery, photodynamic [19] and photothermal [20] therapies, as well as antibacterial agents (Figure 1).

## 5. Bioimaging

Bioimaging includes fluorescent imaging, magnetic resonance imaging (MRI), photoacoustic imaging, and multimodal imaging. CDs exhibit bright photoluminescence and good photostability. Red-emissive tumor-selective CDs (wavelengths between 600 and 700 nm) are desirable for bioimaging and fluorescence-guided surgery due to the reduced autofluorescence and better contrast between tumor and non-tumor tissue [21,22]. Photoluminescence in the red region of visible light was achieved by our group by implementing o-Phenylenediamine (o-PDA) as a precursor and conjugating the resulting CDs to the transferrin ligand [23]. This has been achieved by other groups as well and utilized for imaging in high-acidity environments, analogous to those that might be found in the hypoxic microenvironment at tumor cores [24,25]. NIR-emissive CDs (700–1400 nm) are ideal for in vivo bioimaging applications because NIR light can penetrate deep into tissues [26]. Gadolinium-doped CDs are the most-popular in magnetic resonance imaging (MRI) applications due to their strong contrast enhancement and rapid elimination from the body after MRI examinations [27]. Similarly, highly biocompatible iodine-doped CDs proved to be superior to the conventional computed tomography (CT) contrast agent for contrast-enhanced CT imaging [28].

## 6. Carbon Dot Synthesis

**Figure 2:**
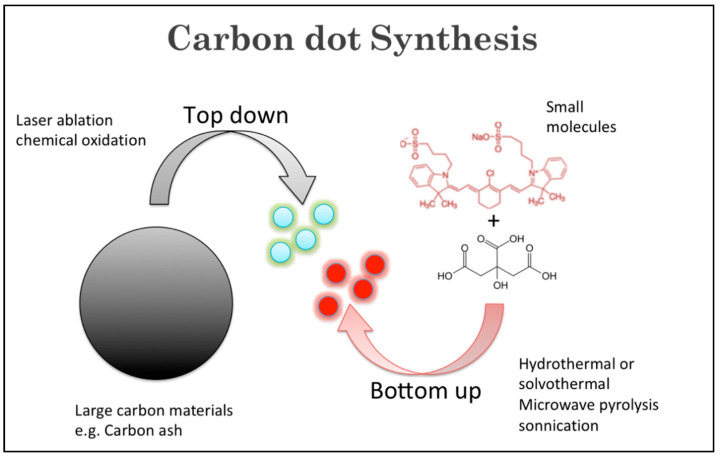
Illustration of top-down and bottom-up CD synthetic approaches.

A wide variety of precursors with strong chemical inertness and numerous surface carboxylic groups can be used to synthesize CDs. The presence of carboxylic moieties results in excellent water solubility. The presence of these groups allows for functionalization with organic, polymeric, inorganic, or biological species. Two synthetic approaches “top-down” and “bottom-up” are common for the preparation of CDs (Figure 2). In the top-down route, a larger carbon structure such as graphite and carbon nanotubes is broken down into CDs using laser irradiation or electrical discharge. In the bottom-up route, small precursors such as carbohydrates and citrate are used to produce CDs through various synthetic techniques such as hydrothermal/solvothermal, microwave, and sonication processes.

The most-popular synthetic approach to prepare CDs is microwave irradiation due to short reaction time, uniform heating, and high yield. Zhu et al. (2009) applied this method for the first time to prepare CDs from polyethylene glycol (PEG) and sucrose [29]. Citric acid is a common precursor in the microwave synthetic process because of its biocompatibility and low cost. Citric acid is often paired with various dopants such as ethylenediamine [30], phenylenediamine [31], and urea [32] to produce a variety of carbon dots. Another popular method to prepare CDs is the hydrothermal/solvothermal method. A wide variety of carbon-containing precursors have been used to synthesize CDs using the hydrothermal or solvothermal method, such as carbohydrates, proteins, and many organic acids including amino acids and citric acids. Zhao et al. used a coplanar compound called 1-[bis(dimethylamino)methylene]-1H-1,2,3-triazolo[4,5-b]pyridinium 3-oxide hexafluorophosphate (HATU) to prepare CDs using a one-step solvothermal method [33]. This method is easy to replicate, and the optical properties can be tuned by modifying the reaction conditions [34]. Less commonly used is ultrasonication, which is the gentlest method, using ultrasonic waves to create very high local temperatures. Zhou et al. (in 2018) reported the synthesis of yellow-emissive CDs (Y-CDs) using the ultrasonication method [35]. The main difference among different CDs resides in the structure, primarily regarding the core. The cores of carbon nanodots are usually amorphous, and each class of CDs has a different core structure. However, their surfaces are believed to be composed of simple functional groups or small organic molecules [36].

**Figure 3:**
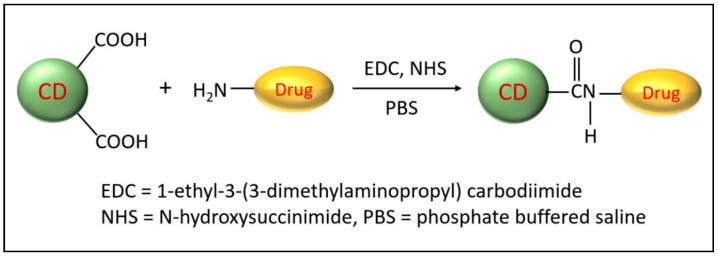
Simplified scheme depicting the conjugation of CDs to a chemotherapeutic agent. Only two CD carboxyl groups are shown for clarification.

Besides the many fascinating optical properties and outstanding biocompatibilities of CDs, unmodified pristine CDs possess some drawbacks, such as poor selectivity and sensitivity toward specific biological systems and low quantum yield. Elemental doping of CDs can greatly modify physiochemical properties including optical properties and surface functional groups and, thus, improve their use in biomedical applications [37]. Likewise, the high surface-area-to-volume ratio allows for efficient modifications via surface passivation and functionalization. Several reviews on the modifications of CDs have recently been published [38,39,40] and, therefore, will only be discussed briefly here. Nanoarchitectonics refers to the creation of functional nanomaterials via the careful application and selection of precursors, synthesis conditions, and surface modifications. In particular, surface modification has generated much attention and can be either accomplished by covalent or non-covalent functionalization. The most-common surface functional groups include carboxyl, hydroxy, and amine and can be utilized to attach tumor-targeting ligands and anti-cancer drugs. The most-common covalent modification utilizes EDC/NHS coupling chemistry to form a stable amide bond between the CD carboxyl group and a primary amine (Figure 3). This is often used to covalently attach a protein or peptide or chemotherapies such as doxorubicin [41] and gemcitabine [42]. Other less-commonly used methods include silylation, esterification, sulfonation, and copolymerization reactions [43]. On the other hand, non-covalent modifications can be achieved by electrostatic, complexation, or π interactions between CDs and the desired molecule. Non-covalent modifications have the unique feature that they do not disrupt the structural integrity of CDs. These modification methods introduce new functional groups and/or target molecules on the surface of CDs, improving their properties and their biological interactions.

## 7. Biocompatibility

Biocompatibility is a key determinant toward the development of nanomaterials intended for biological applications. This is especially true for children since their bodies are still developing, which may make them more vulnerable to adverse effects when utilizing therapies aimed at interfering with cell division. Several preclinical studies have demonstrated the safety and biocompatibility of carbon dots. In 2009, Yang et al. assessed CDs’ biocompatibility for the first time in mice by exposing them to CD aqueous solutions [44]. After four weeks of exposure, the mice demonstrated normal behavior and no negative symptoms. In 2013, the toxicity of various photoluminescent CD concentrations was thoroughly evaluated in mice and rats by biochemical, hematological, and histopathological analyses. No significant toxic effects were observed, and it was concluded that the prepared CDs demonstrated chemical inertness, low cytotoxicity, and good biocompatibility [45]. When tumor-targeted CDs were investigated, it was noted that the CDs preferentially accumulated in the tumor, with negligible organ retention [46]. Furthermore, due to CDs’ small size, they can be rapidly excreted in the urine, unlike other nanomaterials, which can accumulate in the liver and kidneys. The precursors used, route of synthesis, and degree of surface passivation or functionalization could affect how the resulting CD formulation interacts with biological components; therefore, potential toxicities or adverse effects should always be carefully examined.

Our group has published extensively on both the chemical synthesis, as well as preclinical efficacy of CDs used to treat pediatric tumor cell lines. As with other types of nanoparticles, the synthesis greatly dictates their physiochemical properties and, thus, their effect on biological tissues. We recently published our experience with synthesizing CDs from two bottom-up approaches utilizing glucose as a precursor [47]. CDs produced via microwave-assisted synthesis were shown to effectively cross the BBB in zebrafish and rat models, accumulating in neurons. CDs synthesized using hydrothermal carbonization were cytotoxic to pediatric tumor cells specifically. Though both synthesis routes utilized the same precursors and were shown to be non-toxic to non-tumoral cell lines, they resulted in CDs with differing properties and translational utility. The optical properties, as well as functional groups exposed on the CD surface may be selected for during synthesis. CD surface modification via functionalization or passivation can further improve the sensitivity and selectivity for tumor cells, as well as enhance fluorescent intensities. CDs also offer an extremely inexpensive and easy route of synthesis, allowing for large-scale cost-effective reproduction and broad accessibility.

## 8. Crossing the Blood–Brain Barrier

**Figure 4:**
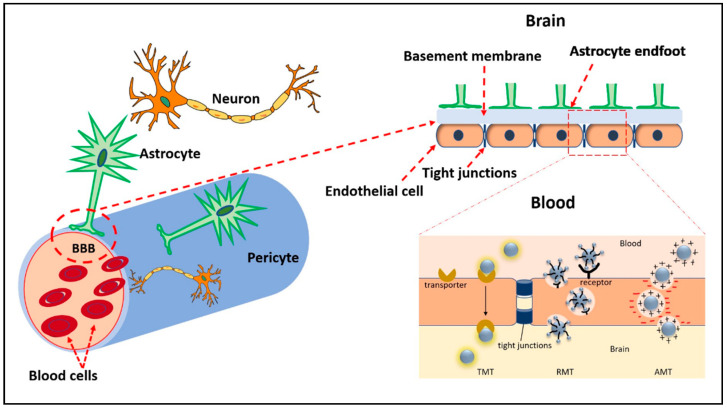
Illustration of the BBB and mechanisms by which CDs may cross. Transporter-mediated transcytosis (TMT), receptor mediated transcytosis (RMT), and adsorptive-mediated transcytosis (AMT).

A major hurdle to effective brain tumor treatment has been the failure of most therapies to cross the BBB at therapeutic levels. The unique microvasculature of the BBB is a structural and functional barrier tightly controlling the entry of molecules into the CNS. Capillary endothelial cells, astrocyte projections, and the basement membrane function as a physical barrier limiting the entry of substances from the circulation into the brain parenchyma. Tight junctions between endothelial cells allow the passage of a few select substances such as oxygen and carbon dioxide (Figure 4). It is estimated that 98% of small molecules and 100% of large molecules cannot cross the BBB, severely limiting the number of therapeutic agents available for brain tumor treatment [48]. Despite this, we and others have demonstrated that both surface-modified and bare CDs can cross the BBB. Using a zebrafish model, Li, S et al. demonstrated that CDs synthesized via the top-down method from carbon powder do not cross the BBB. However, when conjugated to the iron transport protein transferrin, these CDs readily cross the BBB [49]. Transferrin receptors (TfRs) are highly expressed on brain endothelial cells, which makes them excellent targets to transport transferrin-conjugated CDs and other nanoparticles across the BBB via a process known as receptor-mediated transcytosis. While the TfRs are commonly used to facilitate BBB crossing, other receptors present on the BBB can be exploited for transport. For example, angiopep-2, a ligand for the low-density lipoprotein receptor-related protein 1 present on the BBB, can be used to deliver cargo into the brain parenchyma. Liu et al. demonstrated that systemically administered angiopep-2-decorated fluorescent CDs crossed the BBB in healthy Sprague–Dawley rats and accumulated in tumor tissue in a mouse C6 glioma orthotopic brain tumor model [21]. Similarly, CDs can be synthesized to take advantage of transporters expressed at high levels on the BBB such as the glucose transporter GLUT1 or the L-type amino acid transporter 1 (LAT1). Seven et al. demonstrated that fluorescein-conjugated CDs prepared from glucose readily crossed the BBB and were visualized in the CNS by fluorescent microscopy [50]. Furthermore, transporter-mediated uptake was confirmed when yeast expressing glucose transporters showed significantly greater uptake than yeast lacking the transporters. Likewise, Mintz et al. showed that tryptophan CDs readily cross the BBB in a zebrafish model. The authors hypothesized that the residual tryptophan molecules present on the CDs allowed recognition by the LAT1 transporters [51]. Another way CDs can cross the BBB is by adsorptive-mediated transcytosis, which is dependent on electrostatic interactions between negatively charged moieties on the luminal side of brain capillary endothelial cells and positively charged surface functional groups or ligands of the CDs. The methods commonly used to deliver cargo across the BBB are shown in Figure 4.

## 9. Active Targeting

Pediatric tumors in the CNS range from benign inconsequential growths to fast-expanding malignancies. Two tumors that receive the same pathological diagnosis may vary greatly in susceptibility to treatment. Furthermore, it is well established, especially in aggressive tumors such as gliomas, that individual populations of cells within the same tumor may also express different genomic expression profiles, protein expression, and sensitivity to certain treatments. Many chemotherapeutic regiments that target these tumors have peripheral toxicity on unwanted tissues, posing an additional hurdle in treating children specifically. Effective therapies need to account for both inter- and intra-tumoral heterogeneity, underscoring the importance of personalized, real-time molecular analysis and targeted intervention.

To date, most nanomedicines used in the clinic depend on the enhanced permeability and retention effect (EPR). This phenomenon refers to the passive accumulation of nanoparticles within the tumor due to leaky vasculature and reduced lymphatic drainage and has been a key factor in nanomedicine design [52,53]. However, data indicate that very little of the injected dose actually accumulates in the tumor [54]. Two overarching uptake mechanisms have been described in cellular CD uptake. The passive route refers to non-receptor mediated uptake via diffusion or endocytosis of CDs, which has been shown to favor CDs with more positively charged surface moieties, whereas the active route refers to transporter and receptor–ligand-mediated uptake into cells [55,56,57]. CDs can be conjugated to ligands and drug cargo either covalently, via linkers, or by electrostatic conjugation to allow drug release at specific locations through pH release systems, or through reductase-dependent bonds such as disulfide linkage. The targeted tumor, tumor microenvironment, and enzymes present intracellularly must be considered to effectively deliver drug to the target. Understanding the expression landscape of pediatric brain tumor subtypes will aid researchers in the honing of targets for CD-based drug delivery.

Methods to improve tumor cell selectivity primarily involve taking advantage of differentially upregulated membrane receptors, transporters, and antigens, as well as altered tumor cell metabolism. Specifically, tumor cells demonstrate increased glycolysis and amino acid (AA) metabolism. Using nanoarchitectonics, CDs can be generated to target glycolytic or AA transporters, which are expressed at high levels on tumor cells. Furthermore, since the glucose transporter GLUT-1 and the AA transporter LAT1 are present on the BBB, these CDs can cross the BBB for bioimaging and drug delivery purposes.

Brain-tumor-specific CDs were synthesized via thermal hydrolysis using equal molar concentrations of D-glucose and L-aspartic acid. Upon injection into tumor-bearing mice, the fluorescent CDs localized to the tumor in a glioma orthotopic model, confirming that these are in fact brain tumor self-targeting CDs, which can be utilized for brain tumor bioimaging and drug delivery. The authors hypothesized that the CDs contain some reactant functional groups on their surface, which would facilitate their uptake via glucose and AA transporters. Qiao, L. et al. employed nanoarchitectonics to optimize these glioma-cell-targeting CDs by varying ratios of glucose and aspartic acid and found that a ratio of 7:3 demonstrated superior glioma cell selectivity [58]. Using a simple microwave-mediated synthesis method, we generated fluorescent carbon nitride dots (CNDs), using citric acid and urea. Due to carboxylic and primary amine surface functional groups, these CNDs structurally mimic AAs and demonstrate preferential uptake by tumor cells. Treatment of pediatric high-grade glioma (pHGG) and embryonic kidney cells with CNDs covalently conjugated to the chemotherapy gemcitabine induced significantly more cell death in the tumor compared to non-tumor cells. Additionally, both the bare CNDs and chemotherapy-conjugated CNDs readily crossed the BBB when tested in a zebrafish model [49]. Increasing the ratio of urea to citric acid increased the number of primary amine groups, which further increased CND uptake in pHGG cells, as well as increased the chemotherapy drug loading capability. AA transporter inhibitor studies indicated that CND uptake was intricately linked to the upregulated expression of ASCT2 and LAT1 AA transporters on pHGGs [32]. Similarly, Li, S. et al. synthesized CNDs from citric acid and 1,4,5,8-tetraminoanthraquinone (TAAQ) via autoclave-mediated hydrothermal synthesis. The resulting CNDs referred to as large AA-mimicking carbon quantum dots (LAAM CQDs) demonstrated tumor cell selectivity both in vitro and in vivo and, when loaded with the chemotherapy topotecan via π–π-stacking interaction, reduced tumor growth in a mouse orthotopic brain tumor model [46]. Microwave-mediated synthesis of CNDs using citric acid and the anti-diabetic drug metformin resulted in fluorescent CNDs that localize to the nucleus and mitochondria of pHGG cells, but not in non-cancer cell lines [59]. Such CDs would be useful for drugs that work optimally in these organelles.

Alternatively, active targeting can be accomplished by attaching ligands that bind cell surface proteins known to be upregulated in pediatric brain tumors. TfRs are upregulated in tumor cells and present on the BBB, making them an attractive target for nanoparticle-mediated drug delivery [60]. Hettiarachchi, SD, et al. investigated the efficacy of CD-mediated dual-drug delivery and showed that targeted dual-drug delivery (transferrin conjugated) was approximately 100-fold more effective in inducing brain tumor cell death than untargeted CD drug delivery. Furthermore, transferrin-receptor-targeted doxorubicin-conjugated CDs induced more pediatric brain tumor cell death than doxorubicin alone [41]. Bioimaging studies revealed that a pediatric brain tumor cell line treated with the CD conjugate had significantly higher nuclear doxorubicin levels compared to cells treated with doxorubicin alone. However, while transferrin receptors have been utilized as a target for several nanoparticle formulations, they are also expressed on normal cells and, as such, can cause adverse side effects.

The search for cell surface antigens for chimeric antigen receptor (CAR) T-cell therapy for aggressive pediatric brain tumors has identified several candidates for targeted nanoparticle-mediated drug delivery [61]. The most-common antigens for targeting pHGGs, ependymoma, atypical teratoid/rhabdoid tumor, and medulloblastoma are shown in Table 1. With the exception of B7H3, antigen-targeted functionalized nanoparticles have demonstrated anti-cancer effects. To date, targeted drug delivery to B7H3 has been accomplished by an antibody–drug conjugate, but anti-B7H3 antibody targeting can also be applied to nanoparticle-mediated drug delivery.

An alternative strategy for CD-mediated pediatric brain tumor therapy is to target the tumor microenvironment (TME). The TME refers to the cellular and non-cellular components such as low pH and hypoxia, which promote tumor cell proliferation and invasion, immune evasion, and drug resistance. Targeting blood vessel growth with bevacizumab, a VEGF inhibitor has been proven to extend life for GBM patients and has been approved for the treatment of recurrent GBM [72]. Recently, Shoval A et al. demonstrated the anti-angiogenic effects of an anti-VEGF aptamer-modified CD in a mouse ocular disease model [73].

Targeting immune cell components such as activating cytotoxic T-cells with CTLA-4 and PD-1 inhibitors or inhibiting tumor-associated macrophages with colony stimulating factor-1 inhibitors are also promising strategies currently being evaluated as potential therapies for brain tumors in clinical trials. Recently, Su et al. developed PD-L1-targeted CD-based PROTACs, which resulted in PD-L1 degradation and colon cancer cell death [74]. Targeting the integrin family of transmembrane proteins is an attractive target for tumor-specific drug delivery. Integrins are upregulated in cancers and can be expressed on both endothelial and tumor cells including pediatric brain tumors. The RGD peptide (arg-gly-asp) is often used to target integrins. Feng et al. developed a pH-dependent cisplatin drug release integrin-targeted CD and demonstrated significant breast cancer cell death at a lower pH, thereby targeting multiple aspects of the tumor microenvironment [75].

## 10. Future Applications

Though many challenges remain ahead in the optimization of treatment for pediatric brain tumors, exciting advancements have been made in recent years. Refinement of surgical techniques, BBB disruption, targeted drug delivery, and modified radiation therapy have resulted in additional avenues to target and treat these lesions. Combining the therapeutic potential of CDs with these developments may aid in combatting pediatric CNS neoplasms in the future. Currently, histone deacetylation inhibitors and demethylating agents are under investigation as drugs of interest for pediatric brain tumors (Table 2). Conjugating these novel therapies, which show promise with CDs for more effective delivery, may further increase their utility in the future. Chemotherapies that have been used for many years in the clinic, as well as chemotherapies that have proven ineffective historically may be optimized and reinvestigated for their clinical utility when delivered via CDs. The anti-cancer effects of CDs effectively loaded with drugs such as doxorubicin [50], epirubicin [76], gemcitabine [42], topotecan [77], paclitaxel [78], and others has been demonstrated. In addition, anti-cancer agents can be engineered for covalent conjugation to CDs, an area our group is actively pursuing [79,80]. Lastly, CDs can function as radiosensitizers, thereby enhancing the anti-cancer effects of radiotherapy [81]. Furthermore, just as CDs can be conjugated simultaneously to both drug payloads and ligands for targeted delivery, multiple drugs can be carried by CDs to the desired target [76]. Future studies should aim to optimize not only ideal drugs and targeting ligands for conjugation, but also the best combinations and number of payloads to conjugate.

Pediatric neurosurgeons have recently implemented endoscopy to access difficult-to-reach lesions in the brain and spinal canal [87,88]. Though flexible scopes permit tortuous courses to be taken to the target tissue, the use of both rigid and flexible endoscopes has been shown to be safe in endoscopic third ventriculostomy in both pediatric and adult populations [89]. Endoscopy enables the surgeon to minimize the size of the incision and craniotomy, in some cases to a singular burr hole, thereby limiting surgical morbidity. This approach has been used for the treatment of hydrocephalus, intracranial masses, and craniosynostosis. Endoscopic access to the ventricular system would not only enable intraventricular tumors to be removed in a minimally invasive fashion, but also allow for intra-operative delivery of drug into the ventricular system prior to establishing a more permanent route of administration such as an Ommaya catheter. Intra-ventricular delivery of chemotherapy directly into the fourth ventricle has been shown to be relatively well-tolerated, producing a beneficial anti-tumor response in some patients after medulloblastoma resection [90]. More recently, the infusion of methylation inhibitor 5-AZA was also performed directly into the fourth ventricle of pediatric patients with recurrent ependymomas in the posterior fossa without causing neurological toxicity [91].

Aside from direct delivery of therapy into the ventricular system, researchers have sought to deliver drugs systemically and disrupt the BBB surrounding the target lesion. A recent study reviewing medical records from 1997–2019 at one institution demonstrated safe intra-arterial injection of chemotherapy in 12 patients with progressive/and or unresectable pilocytic astrocytoma, for which early radiotherapy could induce long-term neurocognitive deficits [92]. Focused ultrasound is a commonly used, minimally invasive, FDA-approved modality used to induce targeted lesions in certain neuroanatomical regions to treat tremor, for example [93]. Focused ultrasound is also being investigated as a potential therapy to disrupt the BBB near tumors, thereby allowing systematic drug delivery to reach the malignant target cells more easily [94,95]. Radiation is often utilized to treat pediatric CNS malignancy, but toxicity and secondary effects remain a challenge. Proton beam therapy is a modality of particle radiotherapy posited to have better dose localization to the tumor and limited scatter into healthy surrounding tissues, though longer follow-up studies are needed to definitively establish this difference in pediatric patients with CNS tumors [96]. Tumor-treating fields are being used in adult glioma to disrupt chromosomes from effectively lining up at the metaphase plate during mitoses with the aim to slow tumor proliferation [97,98]. It remains to be seen what role this technology may play in pediatric brain tumors and the delivery of nanomedicines to tumors within the CNS.

The ability to specifically target the tumor tissue without affecting normal tissues remains the “holy grail” of cancer treatment. It has been demonstrated that CDs can cross the BBB, localize to tumor tissue, and reduce tumor burden in mouse orthotopic brain tumor models. Combining the recent advances in drug delivery discussed above with CD-mediated drug delivery expands their potential use as drug delivery agents. CDs can be functionalized with multiple tumor-targeting ligands and multiple drugs in order to combat tumor heterogeneity and the development of drug resistance. Molecular profiling has revealed distinct differences between adult and pediatric brain tumors. A better understanding of the disease process and the identification of novel targets and potential new treatment options has paved the way for personalized treatment plans. Since CDs only take a few weeks to synthesize and characterize, it is plausible that targeted CD drug complexes can be prepared on an individual basis for pediatric brain tumor patients.

## 11. Conclusions

Brain tumors remain a significant cause of pediatric morbidity and mortality in the United States and around the globe. Nanoparticles as a therapeutic modality have gained attention in recent years, and CDs in particular offer the benefit of a low cost, high reproducibility, imaging, as well as robust modification for tumor-specific molecular targeting. We posit that the unique properties of CDs make them an excellent choice for pediatric brain tumor therapy, especially compared to other drug delivery systems. For example, liposomes, ranging in size from 50–450 nm, are the most-commonly used drug delivery vehicles in clinical trials [99]. A significant advantage of CDs over liposomes and other nanoparticle drug delivery systems is their small size, a feature that facilitates more rapid and efficient penetration of the BBB, with greater tumor access and dispersion throughout the tumor cells [100,101]. Nanoparticle size is a special concern for children because larger nanoparticles can accumulate in the spleen and liver, increasing toxicity, while smaller nanoparticles are more efficiently cleared via the kidneys [102]. Furthermore, CDs can be easily functionalized with tumor-targeting ligands and various chemotherapies for active tumor cell targeting and killing [41,76]. Specifically, receptor- or transporter-mediated targeting should result in higher tumor cell uptake and reduced off-target effects, which is of utmost importance in treating children. Further pre-clinical research is needed, both on CD characterization and therapeutic optimization, in order to understand the translational utility CDs will have in combatting these malignancies clinically in the future.

## Figures and Tables

**Table 1:** Potential antigens and targeted ligands for CD-mediated drug delivery to pediatric brain tumors.

Targeted Molecule	Function	Natural Ligand(s)	Examples of Targeting Ligand(s) Tested for Nanoparticle Delivery to Tumor Cells
HER2 (ErbB-2)	Receptor	Neuregulin-1 (NRG1)	KCCYSL peptide [62], LTVSPWY peptide [63]
IL13Rα2	Receptor	IL13	Pep-1 [64]
EGFR	Receptor	Several, such as EGF and neuregulin family members, as well as TGF-a	GE11 Peptide [65], anti-EGFR anti-bodies, e.g., Cetuximab [66]
EphA2	Receptor	Several ephrins	Ephrin-A1-mimicking peptide YTPL [67], anti-EphA2-specific antibody fragments [68]
GD2	Ganglioside	Not identified	Anti-GD2 antibody [69,70]
B7H3	Checkpoint inhibitor	IL20RA	Anti-B7H3 antibody [71]

**Table 2:** Epigenetic therapies that are under investigation. Mechanism of action listed, as well as 2D chemical structure for CD conjugation consideration.

Drug Name	Mechanism	Chemical Structure
Panobinostat	Histone deacetylase (HDAC) inhibitor, Partial rescue of H3K27M hypomethylation [82]	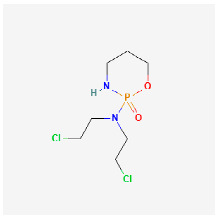
Entinostat	HDAC inhibitor, well tolerated in phase I study in pediatric patients, no dose-limiting toxicity [83]	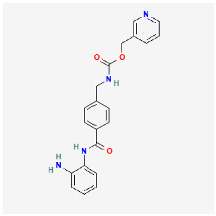
Vorinostat	HDAC inhibitor, well-tolerated in children in combination with temozolomide, dose-limiting myelosuppression [84]	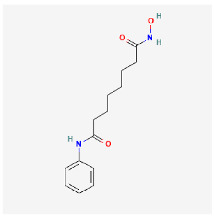
Valproic Acid	Histone hyperacetylation inducer, well tolerated in children with no dose-limiting toxicity [85]	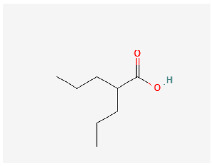
5-aza-2′-deoxycytidine	DNMT methylation inhibitor (demethylating agent), proposed to suppress intratumoral heterogeneity [86]	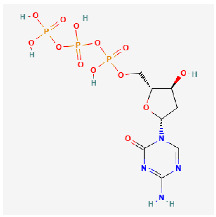

## Data Availability

No new data were created nor analyzed in this study. Data sharing is not applicable to this article.
